# RITA^®^ Temporary Immersion System (TIS) for Biomass Growth Improvement and Ex Situ Conservation of *Viola ucriana* Erben & Raimondo

**DOI:** 10.3390/plants13243530

**Published:** 2024-12-18

**Authors:** Piergiorgio Capaci, Fabrizio Barozzi, Stefania Forciniti, Chiara Anglana, Helena Iuele, Rita Annunziata Accogli, Angela Carra, Marcello Salvatore Lenucci, Loretta L. del Mercato, Gian Pietro Di Sansebastiano

**Affiliations:** 1 Department of Biological and Environmental Sciences and Technologies (Di.S.Te.B.A.), University of Salento, Campus Ecotekne, 73100 Lecce, Italy; piergiorgio.capaci@unisalento.it (P.C.); fabrizio.barozzi@unisalento.it (F.B.); chiara.anglana@unisalento.it (C.A.); rita.accogli@unisalento.it (R.A.A.); marcello.lenucci@unisalento.it (M.S.L.); 2 Institute of Nanotechnology—NANOTEC, Consiglio Nazionale delle Ricerche (CNR), Campus Ecotekne, 73100 Lecce, Italy; stefania.forciniti@nanotec.cnr.it (S.F.); helena.iuele@nanotec.cnr.it (H.I.); loretta.delmercato@nanotec.cnr.it (L.L.d.M.); 3 Institute of Biosciences and Bioresources, National Research Council (CNR-IBBR), Via Ugo La Malfa 153, 90146 Palermo, Italy; angela.carra@ibbr.cnr.it

**Keywords:** *Viola ucriana*, ex situ conservation, Temporary Immersion System (TIS), flow cytometry

## Abstract

*Viola ucriana* Erben & Raimondo is a rare and endangered taxon, endemic to a limited area on Mount Pizzuta in northwestern Sicily, Italy. Its population is significantly threatened by anthropogenic activities, including fires, overgrazing, and habitat alterations. Temporary immersion systems (TISs) have proven effective for large-scale propagation in various protected species, offering potential for ex situ conservation and population reinforcement of *V. ucriana*. This study aimed to establish a bioreactor-based micropropagation protocol for shoot multiplication and compare the efficacy of a TIS with that of conventional solid culture medium (SCM). Three different plant growth regulators (PGRs) were also compared: 6-benzylaminopurine (BA), zeatin, and meta-topolin-9-riboside (*m*TR). The starting material originated from seeds collected from mother plants in their natural environment. The best growth outcomes (in terms of shoot multiplication, shoot length, and relative growth rate) were achieved using THE RITA® TIS, with BA (0.2 mg/L) and mTR (0.5 or 0.8 mg/L) outperforming SCM. Anomalous or hyperhydric shoots were observed with all zeatin treatments (especially with 0.8 mg/L) in both the TIS and SCM, suggesting that this cytokinin is unsuitable for *V. ucriana* biomass production. The rooting phase was significantly improved by transferring propagules onto rockwool cubes fertilized with Hoagland solution. This approach yielded more robust roots in terms of number and length compared to the conventional agar-based medium supplemented with indole-3-butyric acid (IBA). Flow cytometry analysis confirmed the genetic fidelity of the regenerants from the optimal PGR treatments, showing that all plantlets maintained the diploid ploidy level of their maternal plants. Over 90% of the in vitro derived plantlets were successfully acclimatized to greenhouse conditions. This paper represents the first report of *V. ucriana* biomass multiplication using a RITA^®^ bioreactor. The stability of the regenerants, confirmed by nuclei quantification via cytofluorimetry, provides guidance in establishing a true-to-type ex situ population, supporting conservation and future reinforcement efforts.

## 1. Introduction

The *Violaceae* family comprises perennial, annual, herbaceous, and shrub plants primarily distributed in temperate–mountainous regions. The largest genus within this family is *Viola* L., which includes 46 species in Italy, of which 18 are endemic [1]. In Sicily, *Viola* sect. *Melanium* consists of six species [2]. Among them, *Viola ucriana* Erben & Raimondo, along with 15 other species and subspecies, belongs to the *Calcarata* species complex [3,4,5,6].

The *Calcarata* complex is well known for its distinctive morphological features, such as large flowers; dimorphic leaves; pinnately or palmately divided stipules; and long, thin ascending primary axes. Originally, *V. ucriana* was considered a variety of *Viola nebrodensis* C. Presl, along with *Viola tineorum* Erben & Raimondo. However, *V. ucriana* and *V. tineorum* were later elevated to the species rank due to significant differences in their anatomical characteristics, including leaf micromorphology, as well as stem, petiole, and seed structure [2,3].

From an ecological point of view, *V. ucriana* grows near the summit of Mount Pizzuta, (Sicily, Italy), the highest point in the Palermo Mountains [6]. It thrives in calcareous-dolomitic soil, preferring sunny cavities within a patchwork of garrigue dominated by *Erica multiflora* L., and in perennial meso-xerophilus grassland characterized by *Brachipodium rupestre* (Host) Roem. & Schult and *Ampelodesmos mauritanicus* (Poir) T. Durand & Schinz [7]. *V. ucriana* is strictly linked to the *Lolio pluriflori-Brachypodietum* vegetation type [8].

According to the International Union for Conservation of Nature (IUCN), this species is classified as Critically Endangered (CR) due to its extremely limited distribution, with the only known population confined to an area of just 8 km^2^ [7]. The precise number of mature individuals and key aspects of its reproductive biology remain unknown. Furthermore, the population is in decline, primarily due anthropogenic pressures. The increasing frequency and intensity of fires during the flowering and seed dispersal periods have had detrimental effects on both sexual reproduction and the species’ ability to colonize new areas [8]. The introduction of non-native pine and broadleaf tree species to grassland areas have been further altering its habitat. Additionally, pastoral activities have exacerbated the challenges facing the species’ conservation.

Currently, *V. ucriana* is not protected by law or international convention agreements, and in situ conservation efforts have proven ineffective, as all known population live outside of the “Serre della Pizzuta” Nature Reserve [7]. In addition, there is a lack of information regarding optimal seed germination protocols and storage conditions for *Viola* species, limiting conservation and restoration efforts through seed banks. This challenge is further compounded by the difficulty in collecting wild seeds, as mature fruit capsules disperse seeds ballistically, often over distances of at least 5 m from the parent plant, within a short seasonal window [9].

Species within *Viola* sect. *Melanium* generally exhibit physiological dormancy, requiring pretreatments such as cold stratification and/or hormone application to break dormancy and promote germination [10]. Given all the issues associated with both the ex situ and in situ protection of *V. ucriana*, along with the limited scientific knowledge of its germination requirements, developing an in vitro plant multiplication method is essential for its conservation.

Micropropagation offers an effective technique for biodiversity conservation and restoration, enabling the preservation of valuable genetic resources [11]. Although tissue culture methods (such as the semisolid system) can be more expensive than conventional propagation, they are fundamental for achieving rapid conservation outcomes for some species [12]. Methods developed with recent advances in *in vitro* culture, particularly with the use of the temporary immersion system (TIS), have demonstrated significant advantages over both solid and liquid systems. These benefits include streamlined scalability and automation, improved accessibility of the medium’s components to the explants, and easier handling [13]. TIS technology also reduces labor requirements; enhances biomass proliferation; and mitigates issues such as malformations, asphyxia, and hyperhydricity [14].

In particular, the RITA^®^ (Recipient à Immersion Temporaire Automatique) bioreactor (CIRAD, St-Mathieu de Tréviers, France, VITROPIC) is a TIS system used for intensive micropropagation and is now being applied for conservation and restoration, surpassing the effectiveness of conventional methods [12].

Many tissue culture protocols have been developed for other *Viola* species via indirect organogenesis. For example, *V. baoshanensis* has been propagated using leaf, petiole, and root tissues [15]; *V. odorata* L. using leaves, stem, and petioles [16]; *Viola patrinii* (Ging) DC. [17] using petioles; *Viola stagnina* Kit. [18] using leaves with petioles; *Viola uliginosa* Besser [19] using petioles and leaves; *Viola serpens* (Ging) Wall. [20] using leaf primordial tissues,; *Viola arvensis* Murray [21] using leaves; *Viola canescens* Wall. [11] using cotyledonary nodes. In a few cases, direct organogenesis was achieved with *Viola* species. For *Viola pilosa* Lam. [22], new individuals were obtained using axillary buds; for *Viola tricolor* L. [23], using shoots; and for *Viola palustris* L. [12], using shoot clumps. However, when using highly differentiated explants such as roots, leaves, petioles, internodes, cotyledons, hypocotyls, and anthers for in *vitro* multiplication, the approach may lead to somaclonal variation [24]. For germplasm preservation, explants like shoots or axillary buds with pre-existing meristems are generally preferred [24] as they are less likely to induce genetic instability. The in vitro regeneration of plants with altered genotypes could compromise the genetic integrity of protected species, making them unsuitable for use in ex situ conservation programs [25].

Several tools are available for the detection of somaclonal variation such as the identification of differences in morphological traits [26], the analysis of chromosomes [27], biochemical studies [28], or using molecular DNA markers [29]. Among them, flow cytometry is considered a rapid and efficient technique for estimating DNA content and for determining ploidy in plants cultivated in vitro [30].

In this study, we applied the RITA® TIS system for the in vitro multiplication of the rare violet species *V. ucriana*, which is classified as Critically Endangered. The results of this research contribute to the conservation of this species by establishing an ex situ population in the Botanical Garden of Salento. A novel approach for extracting and quantifying nuclei using protoplasts—rather than direct isolation from leaves—was developed. Additionally, flow cytometry was employed to assess the genetic stability and confirm the ploidy levels of the in vitro derived plants, ensuring the conservation of a true-to-type population.

## 2. Results

### 2.1. In Vitro Seed Germination and Plantlets Cultivation

The germination test was carried out on 80 carefully selected seeds of *V. ucriana* by applying cold stratification (5 °C/90 days) and supplementing the MS medium with 250 mg/L of GA_3_. No germination was recorded after 30 days of culture. However, after 60 days, 28 seeds germinated, resulting in a germination rate of 35%. Prolonged culture on MS medium with GA_3_ under chilling conditions increased the germination rate to 63.75% (51 seeds germinated) (Supplementary Figure S1). After 90 days, no further germination was observed.

The seedlings were transferred to MS medium supplemented with 0.1 mg/L BA to obtain sufficient shoots for the experiments. Multiple shoots generated from a single seedling were separated into single 2 cm shoots and subcultured weekly on the same medium. This step was repeated for 12 weeks. Once the desired number of shoots was obtained, comparative experiments were initiated in a solid culture medium (SCM) system and the RITA^®^ TIS. Shoot multiplication was stimulated using pre-existing meristems (single shoots) from multiple rounds of seedling multiplication on MS medium supplemented with 0.1 mg/L of BA.

### 2.2. Effect of Culture System and PGRs on Shoot Multiplication and Shoot Length

**Figure 1:**
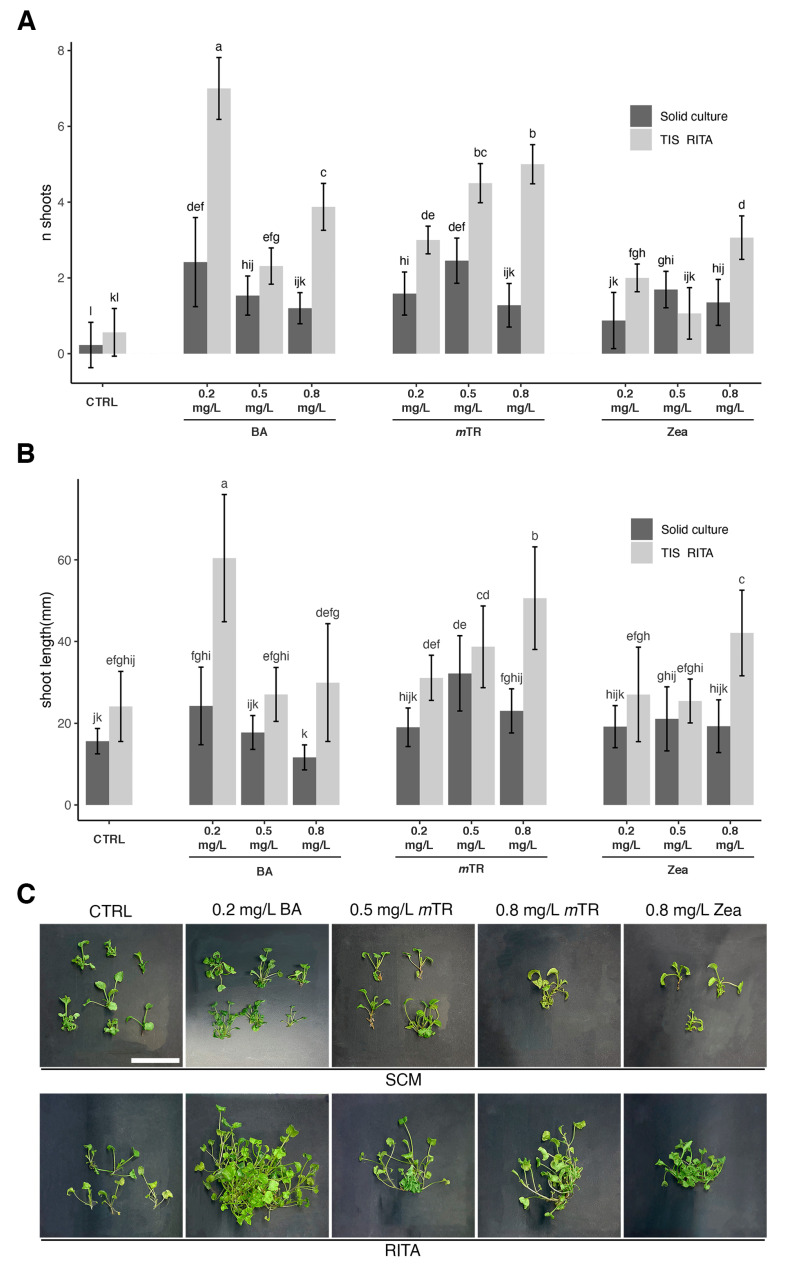
Effect of culture system and PGR (0.2–0.5–0.8 mg/L BA, 0.2–0.5–0.8 mg/L *m*TR, 0.2–0.5–0.8 mg/L zeatin) on shoot multiplication and shoot length of *V. ucriana* after 4 weeks of culture. (**A**) Differences in number of shoots produced in SCM and RITA® TIS with different PGR concentrations. (**B**) Differences in shoot length produced in SCM and RITA® TIS with different PGR concentrations. (**C**) Selection of images showing morphological appearance of shoot multiplication and shoot length after 4 weeks of culture with best treatment (0.2 mg/L BA, 0.5 mg/L *m*TR, 0.8 mg/L *m*TR) and worst treatment (0.8 mg/L zeatin). Scale bar = 25 mm. Statistical analysis with two-way ANOVA with Tukey’s post hoc test (*p* < 0.001). Different letters within bars indicate significant differences. Data presented as mean values ± standard error of three replications of 30 explants each for both culture systems.

The shoot multiplication of *V. ucriana* started from 2 cm shoot explants for both the SCM and RITA^®^ TIS. Significant differences were observed among culture systems and PGR concentrations. The RITA^®^ TIS appeared to be more efficient in producing new shoots, regardless of the PGR used. Even under control conditions (Figure 1A), the RITA^®^ TIS formed more shoots (0.56 ± 0.69) than SCM (0.22 ± 0.59).

The highest shoot numbers in RITA® TIS occurred with 0.2 mg/L BA (7 ± 0.81), followed by 0.8 mg/L *m*TR (5 ± 0.51) and 0.5 mg/L *m*TR (4.5 ± 0.51). The lowest shoot multiplication in RITA® TIS was observed with 0.5 mg/L Zeatin (1.06 ± 0.68). In SCM, the best results were achieved with 0.5 mg/L *m*TR and 0.2 mg/L BA, yielding 2.45 ± 0.59 and 2.41 ± 1.17 new shoots, respectively. The lowest performance was seen with 0.2 mg/L Zeatin (0.87 ± 0.74 new shoots)

The response to the PGRs varied between culture systems. For BA, higher concentrations reduced shoot formation, with low concentrations being the most effective, especially in the RITA^® TIS^. Using *m*TR, the intermediate concentration (0.5 mg/L) was optimal in the SCM, whereas in the RITA^®^ TIS, shoot induction increased proportionally with *mTR* concentration. Zeatin had the poorest overall performance in both culture systems, with inconsistent effects, thus requiring further investigation (Figure 1A).

Shoot length also differed significantly between culture systems and PGR treatments (Figure 1B). Under control conditions, the RITA® TIS produced longer shoots (24.13 ± 8.58 mm) than the SCM (11.65 ± 3.05 mm). In the RITA^®^ TIS, the longest shoots were achieved with 0.2 mg/L BA (60.41 ± 15 mm), 0.8 mg/L *m*TR (50.60 ± 12.57 mm), and 0.8 mg/L zeatin (42.10 ± 10.45 mm). A good result was also observed with 0.5 mg/L *m*TR, with which the length of the shoots reached 38.71 ± 9.98 mm. In the SCM, the shoot length was stimulated to a lesser extent. The longest shoots were observed with 0.5 mg/L *m*TR (32.21 ± 9.19 mm), followed by 0.2 mg/L BA (24.26 ± 9.50 mm) and 0.8 mg/L *m*TR (23.04 ± 5.40 mm).

Shoot length and shoot number were similarly influenced by the PGRs. High BA doses were detrimental to shoot length, whereas increasing the *m*TR concentration had a positive impact. The effect of zeatin was inconsistent: it appeared ineffective in the SCM but produced some elongation in the RITA® TIS (Figure 1B).

Regarding shoot appearance, callus formation was not observed in any of the PGR treatments or culture systems tested. Shoot development was widely different among the different PGRs and culture systems applied (Supplementary Figure S2). Shoots grown without PGRs were similar in terms of vigor, length, and leaf morphology in both culture systems. In the SCM, the healthiest shoots were observed with 0.2 mg/L BA and 0.5 mg/L *m*TR, which displayed proper morphology and continuous proliferation (Figure 1C). While 0.8 mg/L *m*TR also produced healthy shoots, higher concentrations led to the slight yellowing of the stems and leaves. Shoots cultured with 0.8 mg/L zeatin remained undeveloped, with small vitrified buds at the basal part. In all the treatment replicates, the plants were hyperhydric, the shoots had smaller and atrophic stems, and the leaves had structural anomalies (deformation or curling).

In the RITA^®^ TIS, well-developed new shoots were observed with 0.2 mg/L BA and intermediate-to-high *m*TR concentrations (Figure 1C). In these treatments, shoot regeneration showed a remarkable improvement in comparison with that in the SCM (Figure 1A), with no signs of hyperhydricity observed (Figure 1C). Moreover, the number of shoots produced was larger, the stems were noticeably larger in both diameter and length (Figure 1B), and the leaves had the correct morphology. Overall, the plant proliferation efficiency in the RITA® TIS was more efficient than in the SCM.

Also, in the RITA® TIS, supplementation with zeatin at 0.8 mg/L caused the vitrification of the explants (Figure 1C), despite the improvement in shoot regeneration that was observed when compared with the SCM (Figure 1A). These shoots did not survive rooting.

### 2.3. Effect of Culture Systems and PGRs on RGR Index

**Figure 2:**
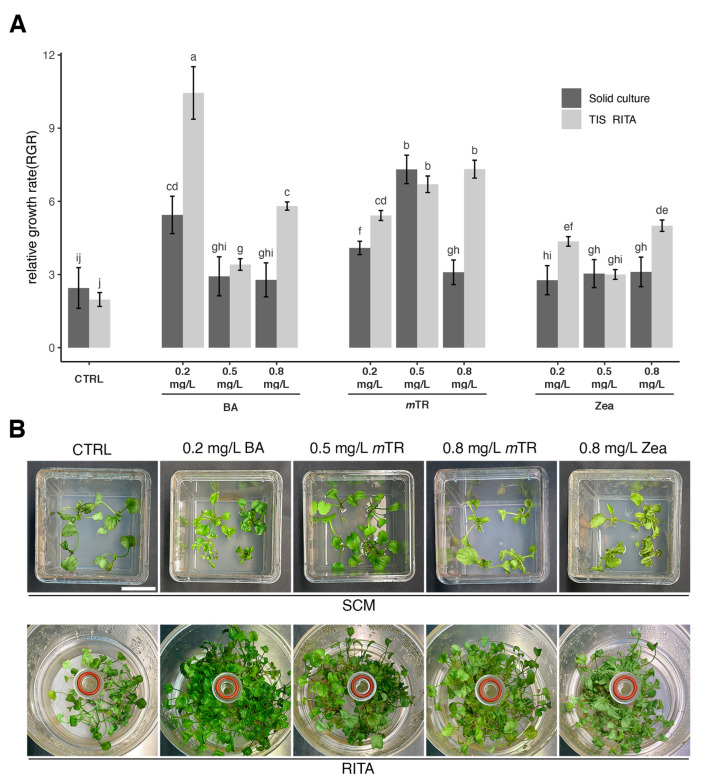
Effect of culture system and PGR (0.2–0.5–0.8 mg/L BA, 0.2–0.5–0.8 mg/L *m*TR, 0.2–0.5–0.8 mg/L Zeatin) on relative growth rate (RGR) of *V. ucriana* after 4 weeks of culture. (**A**) Differences in RGR index in SCM and RITA® TIS with different PGR concentrations. (**B**) Comparison between biomass produced with SCM and RITA® TIS with the best treatment (0.2 mg/L BA, 0.5 mg/L *m*TR, 0.8 mg/L *m*TR) and the worst one (0.8 mg/L zeatin) in terms of hyperhydric and unviable explants. Biomass appearance after 4 weeks of culture. Scale bar = 25 mm. Statistical analysis was conducted with two-way ANOVA with Tukey’s post hoc test (*p* < 0.001). Different letters within bars indicate significant differences. Data are presented as mean values ± standard error of three replications of 30 explants each for both culture systems.

The relative growth rate (RGR) index was used to reflect the effects of the PGRs on various parameters, and no significant effect was observed due to the cultivation system. Under control conditions, the RITA® TIS and SCM were not significantly different (2.44 ± 0.83 vs. 1.97 ± 0.28, respectively) (Figure 2A).

The RITA® TIS enhanced the RGR in combination with the growth regulators, showing the best performance (10.44 ± 1.07) with 0.2 mg/L BA. Increasing the concentration of *m*TR correlated to the RGR value but zeatin did not show a clear trend. In the SCM, low BA concentrations positively affected the RGR, but the best effect, better than that in the RITA® TIS, was observed when 0.5 mg/L *m*TR was applied (7.30 ± 0.58). The zeatin concentration did not have any effect (Figure 2A).

The biomass growth varied with the culture system, type of PGR, and PGR concentrations (Supplementary Figure S3). Figure S3 shows the effect of the treatments in stimulating biomass growth. Observing the CTRL condition in the SCM, the biomass was characterized by limited development. An improvement was obtained by adding 0.2 mg/L BA or 0.5 mg/L *m*TR. Increasing the *m*TR concentration (0.8 mg/L) caused the biomass and number of leaves to reduce as well as to change color to yellow. The higher zeatin concentration (0.8 mg/L) led to hyperhydric symptoms, incorrect morphology, and wrinkled leaves with a swollen, brittle, and yellow appearance (Figure 2B).

In the RITA® TIS, biomass growth was more vigorous. In the CTRL, the biomass growth was healthier and more elongated compared to in the SCM. The 0.2 mg/L BA treatment induced an outstanding micropropagation rate, in which biomass growth was vigorous as well as morphologically perfect and dense and was characterized by abundant and elongated shoots. Similar results were obtained with 0.5 mg/L and 0.8 mg/L *m*TR, with some differences in leaf color (slightly yellow) and minor shoot density. However, biomass was viable and the leaves did not show morphological defects.

Using zeatin, the RITA® TIS exacerbated the morphological disorders observed in the SCM. Overall, the biomass was hyperhydric and unviable, with yellow–grey leaves that were morphologically different from those in the CTRL.

### 2.4. Rooting Phase in SCM, Rooting Plugs, and Acclimatization

Significant differences were observed between the SCM (with or without IBA) and the rooting plug systems in the number and length of the roots produced.

**Figure 3:**
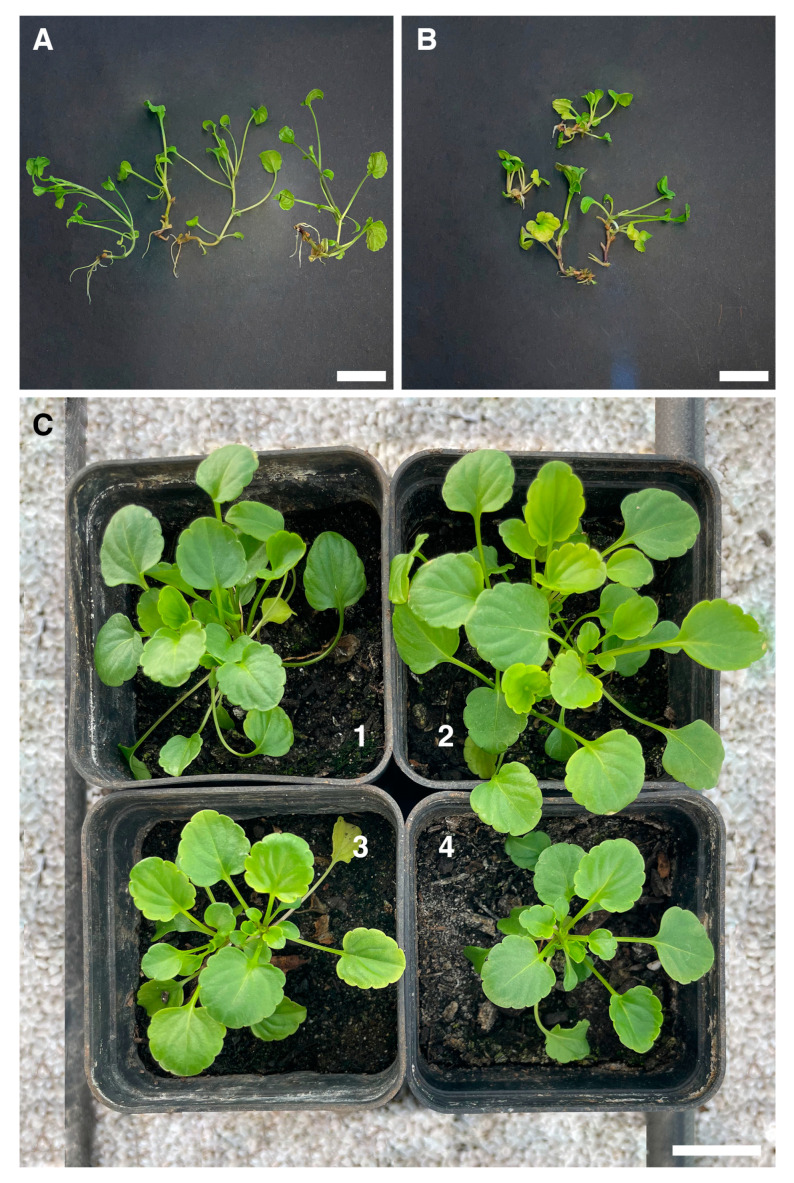
Differences in number and length of roots grown (**A**) in rooting plugs with rockwool cubes and (**B**) in ½ MS medium with 2 mg/L IBA after 5 weeks. (**C**) Plantlets 3 months after potting up from rooting plugs (**C1**, **C2**) and from MS medium with 2 mg/L IBA (**C3**, **C4**) located int the Unisalento Botanical Garden greenhouse. Scale bars (**A**,**B**) = 10 mm. Scale bar (**C**) = 25 mm.

After 5 weeks, more roots were produced using the rockwool system (Figure 3A) (2.53 ± 1.24) and they were longer (22.39 ± 2.16 mm) than those produced in ½ MS medium supplemented with 2 mg/L IBA (Figure 3B), in which the mean number was 1.13 ± 0.83 and the length was 10.73 ± 1.92 mm (Supplementary Figures S4 and S5).

No root formation was observed in the plantlets cultivated on SCM with PGRs either without ½ MS medium or in ½ MS medium supplemented with 0.5 mg/L IBA and 1 mg/L IBA.

Acclimatization was highly successful, with 95% plant survival in the greenhouse of the Botanical Garden of the University of Salento (Figure 3C).

### 2.5. Ploidy Level Determination

**Figure 4:**
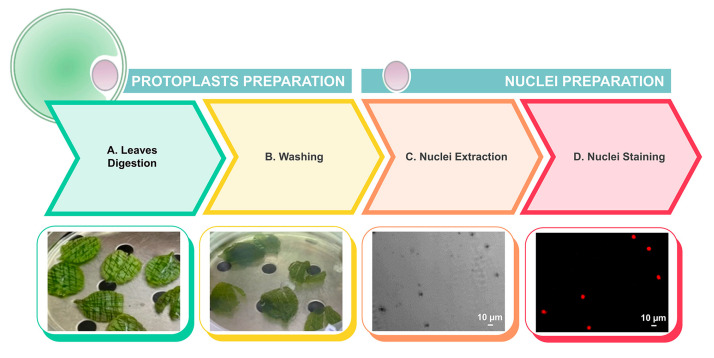
Optimization of nuclei isolation method using leaves of *V. ucriana*. (**A**) Leaves were digested for 18 h using a digestive solution. (**B**) Protoplasts were separated from debris (mucilage, phenolic compounds, DNAse, RNAse, etc.), and the solution was washed with W5 buffer. (**C**) Nuclei were extracted from protoplasts using nuclei isolation buffer. (**D**) Nuclei were stained with propidium iodide (PI) before being analyzed with flow cytometry. Representative CLSM micrographs showing *V. ucriana* nuclei. BF and red channel (propidium iodide: λex 543 nm, λem 570–700 nm). Scale bars: 10 μm.

Due to the large quantities of mucilaginous compounds in the cytosol of the leaf cells, isolating suitable nuclei for flow cytometry required protoplast preparation through a series of optimized steps, as illustrated in Figure 4.

**Figure 5:**
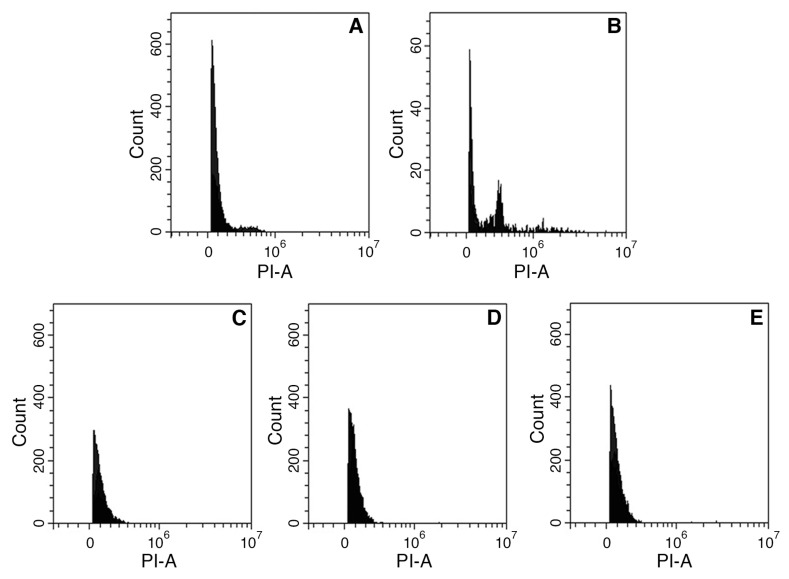
Flow cytometry analysis for the determination of DNA ploidy level. Nuclei were stained with PI indicating the DNA content from *A. thaliana* (**A**) and *V. ucriana* originating from (**B**) mother plants, (**C**) 0.2 mg/L BAP, (**D**) 0.5 mg/L *m*TR, (**E**) 0.8 mg/L *m*TR. In all histograms, peaks represent 2C DNA content of plant materials.

The flow cytometry histograms of the nuclear DNA content (Figure 5) showed the presence of nuclei in the G0/G1 phase of the cell cycle. A secondary peak (Figure 5B) corresponding to the nuclei in the G_2_ phase was more prominent in the mother plants, likely due to the greater maturity of their tissues. All in vitro derived plants regenerated from the best treatments exhibited a 2C DNA content, confirming their diploid status as the mother plants.

## 3. Discussion

In this study, an efficient micropropagation protocol for *V. ucriana* was established for the first time comparing two culture systems and evaluating three concentrations of three different cytokinins (BA, *m*TR, and zeatin).

The plant material was obtained from seeds collected from nature. Previous research by Kilgore et al. [9] demonstrated that cold stratification is the most effective seed pretreatment for breaking physiological dormancy in violet species, whereas GA_3_ can further enhance the seed germination rates. These findings were supported by those of the present study. Magrini and Zucconi [10] also confirmed physiological dormancy in four closely related European pansies occurring in Italy and belonging to Sect. *Melanium* as *V. ucriana.* Notably, no germination was observed without cold stratification in two of these species (*V. arvenisis* subsp. *arvensis* and *Viola kitaibeliana* Shult.). Similarly, in *Viola cornuta* L. ‘Lutea Splendens’, the germination rates improved to 71.2% under cold conditions (10 or 4 °C) [31]. Here, cold stratification and GA_3_ application yielded a germination rate of 63.75%. The shoots derived from the germinated seedlings were used for micropropagation experiments. The number of shoots, shoot length, and RGR index were analyzed in both SCM and RITA^®^ systems to determine the optimal micropropagation rate. Assessing the growth parameters was crucial for determining the optimal combination of culture system and cytokinin concentration, ultimately achieving suitable biomass productivity for the conservation of *V. ucriana*.

It was found that the shoot explants showed great adaptability when cultivated in the RITA® TIS, and the analyses of the growth parameters clearly demonstrated that *V. ucriana* plant efficiency was significantly higher in the RITA® TIS compared to in the conventional SCM approach. Our results showed that the RITA® TIS is preferable to SCM for the large-scale micropropagation and conservation of the endangered violet *V. ucriana*.

New in vitro techniques such as TIS bioreactors have been successfully applied with different plants to improve mass propagation efficiency. TIS efficiency has been reported for rare orchids [32,33,34], horticulture and medicinal plants [35], woody plants [13,36], and protected species [37].

In the *Viola* genus, TIS technology was only previously applied for *Viola palustris*, in which it was also demonstrated that the RITA^®^ bioreactor together with a low concentration of thidiazuron (0.15 mg/L) significantly improved multiplication [12].

The efficiency of the TIS can be attributed to the optimal microenvironmental conditions, which enhance nutrient uptake and gas exchange. Some morphological disorders, such as hyperhydricity, were reduced with the proper adjustment of the immersion frequency and duration [14].

The TIS method could also be applicable for the production of valuable secondary metabolites such as bioactive alkaloids in *Pancratium maritimum* L. [38]; for the rapid micropropagation of species useful for phytoremediation purposes, such as enabling *Dittrichia viscosa* (L.) Greuter lines to tolerate As [39] or aquatic mosses known to absorb several trace elements [40]; and for the production of valuable recombinant proteins such as the modified form of the green fluorescent protein and a vaccine antigen: fragment C of the tetanus toxin by tobacco (*Nicotiana tabacum* L. cv. Petit Havana) transplastomic shoots [41]. The PGRs influenced the growth rate in a similar manner in both culture systems; however, the RITA® TIS enhanced their positive or negative effects on the shoots. It was observed in this species that a lower concentration of BA was needed in the RITA^®^ bioreactor (0.2 mg/L) to improve the micropropagation rate and obtain shoots without morphological disorders. Here, for the first time, we tested *m*TR in the genus *Viola* spp., finding a positive effect on shoot multiplication, especially when applied at the highest concentration (0.8 mg/L). Higher doses of *m*TR should be tested in the future to determine whether overdosage leads to vitrification or enhances plant production.

In the RITA® TIS, 0.2mg/L BA, 0.5 mg/L *m*TR, and 0.8 mg/L *m*TR exhibited good performance in terms of both shoot multiplication and shoot length.

The role of zeatin was unclear, but it is certainly not ideal for this plan material. It exhibited low performance in terms of shoot multiplication in both culture systems. In addition, the high concentration of zeatin (0.8 mg/L), in comparison with the other cytokinins tested, caused hyperhydric symptoms, and the biomass obtained was not viable or usable for the rooting and acclimatization phases.

It is known that high doses of cytokinin induce hyperhydricity. This phenomenon is dose-dependent [42,43] and could be more evident with zeatin or BA [43].

With regard to the PGRs’ effects on the RGR index, rising concentrations of BA were inversely correlated with biomass production when applied in both the RITA® TIS and SCM. Differences in performance were observed with *m*TR stimulation. In the RITA® TIS, rising doses improved the RGR; on the contrary, in the conventional SCM method, the intermediate dose appeared more suitable for improving performance, being comparable to the TIS.

The ultimate goal of this study was to conserve *V. ucriana* while preserving its genetic fidelity. The genetic fidelity of regenerants is better maintained when tissue with preformed meristems is used in micropropagation such as single-double shoots, as we did in the present work. However, somaclonal variation induced by culture conditions cannot be entirely excluded, and a control is necessary, especially when the aim is the ex situ conservation, protection, or reintroduction of rare and/or endangered species [19]. Among the available techniques, flow cytometry was employed for ploidy determination due to its efficiency, cost-effectiveness, and reliability compared to those of chromosome counting [44].

A modified nuclei isolation protocol was developed starting from protoplasts extracted from the leaf tissue of *V. ucriana.* The standard nuclei isolation buffer used for *V. uliginosa* [19] and recommended for mucilaginous species [45] was not suitable for this species. The abundant mucilaginous compounds (observed during leaf chopping with a razor blade) present in the cytosol of *V. ucriana* leaf cells (mother and regenerated plants) prevented efficient nuclei isolation. Indeed, slime production as well as phenolic compounds can negatively affect nuclei isolation and staining (i.e., PI accessibility to DNA) leading to errors in DNA content estimation [30,46].

To address these issues, protoplasts were produced, followed by intact nuclei isolation using a hypotonic buffer [47]. While this approach improved the results, protoplast preparation inherently reduces cell availability. To mitigate this, we used three times the weight of the fresh material from the mother plant (with more mature and expanded leaves) compared to regenerated plants, compensating for potential losses in nuclei recovery. PI staining confirmed the effectiveness and cleanliness of the nuclear suspension. Despite using more material, nuclei recovery was lower from that mother plants compared to that from the regenerated plantlets. This difference could be further explained by the presence of a small number of more expanded cells with a higher cell wall biomass. Conversely, higher cell division occurs in younger plant tissues, leading to a larger cell content and consequently higher nuclei recovery [48,49]. In addition, the mother plants showed a detectable G_2_ peak, typically observed in older and dormant leaves, as reported by Dolezel et al. [47]. The flow cytometry histograms confirmed consistent diploid ploidy levels between the mother and regenerated plants.

In conclusion, this study developed a robust in vitro micropropagation protocol using the RITA® TIS bioreactor for the ex situ conservation of *V. ucriana* and optimized a flow-cytometry-based method for the genetic stability assessment of this plant species. This method provides mucilage-free nuclei for ploidy detection, overcoming the limits imposed by secondary metabolites and confirming a true-to-type population for future reinforcement.

## 4. Materials and Methods

### 4.1. Seed Collection, Sterilization, and Pretreatment

Seeds of *V. ucriana* were collected in June 2022 on Pizzuta Mount (Piana degli Albanesi, NW Sicily, Italy) at five different sites (Supplementary Figure S6). Only 5 ripe fruit capsules with approximately 80 seeds were harvested due to the lack of seed availability in the wild (few mature individuals were found, and ballistic dispersion of seeds made seed harvesting challenging) and in order to avoid a negative impact on the limited population. Fruit split open with seeds ready to be ballistically dispersed and fruit pointed upward (Figure S7A) were selected, as suggested by Kilgore and colleagues [9]. After collection, the seeds were placed in closed Petri dishes to prevent seed loss and ensure they reached maturity (Figure S7B,C). Seeds were surface-sterilized in a 5% solution of commercial bleach + few drops of Tween 20 for 5 min, followed by 3 rinses in sterile distilled water for 10 min. Then, sterilized seeds were rinsed with 3% (*v*/*v*) Plant Preservative Mixture ™ (PPM™, Plant Cell Technology, Washington, DC, USA) for 30 min and left to dry under a laminar flow bench.

Seeds were germinated following the pretreatments carried out by Magrini et al. [10], in which seeds were subjected to cold stratification (5 °C/90 days) in the presence of GA_3_ (250 mg/L) in MS medium.

Sterilized seeds were cultured on MS medium [50] supplemented with 30 g/L sucrose, 250 mg/L GA_3_, and 0.1% PPM™, and gelled with 0.7% (*w*/*v*) agar (Duchefa Biochemie, Haarlem, The Netherlands) for 12 weeks. The pH of the culture media was adjusted to 5.8 using 1 M KOH before autoclave sterilization. In each Petri dish, 16 seeds were placed in 5 replicates, sealed with Parafilm^®^, and covered with aluminum foil. Subsequently, seeds were transferred to a cold chamber at 5 °C/90 days for cold stratification.

The number of seeds germinated was determined after periods of 4, 8, and 12 weeks. The percentage of germination was calculated based on the total number of seeds germinated after 12 weeks, divided by the total number of seeds collected × 100.

### 4.2. Shoot Multiplication from Seedlings on Agar Medium

Seedlings with three fully developed leaves were transferred in full-strength MS medium with 30 g/L sucrose, 0.1% PPM, and 0.1 mg/L of BA and solidified with 0.7% (*w*/*v*) agar (Duchefa Biochemie, Haarlem, The Netherlands). Subculturing of shoots was carried out monthly for 12 weeks in order to obtain sufficient explants for further comparative experiments on RITA® TIS and SCM. The repeated round of multiplication on this medium was conducted in Magenta™ GA-7 vessel (Merk Life Science S.r.l., Milano Italy). Shoots were maintained in a culture room at a constant 25 ± 2 °C under a 16 h photoperiod (60–90 µmol photons m^−2^ s^−1^, cool white fluorescent light).

### 4.3. In Vitro Shoot Multiplication on RITA® TIS and SCM

The SCM and RITA® TIS in vitro culture systems were compared. In the SCM system, shoots were cultured in Magenta™ GA-7 vessels (Merk Life Science S.r.l., Milano, Italy) on full-strength MS medium with 30 g/L sucrose, 0.1% PPM, and 0.7% (*w*/*v*) agar (Duchefa Biochemie, Haarlem, The Netherlands). In addition to the control (CTRL) condition, the plant growth regulators (PGRs) 6-Bbenzylamino-purine (BA), meta-topolin riboside (mTR), and zeatin (Zea) were tested separately at three different concentrations (0.2 mg/L, 0.5 mg/L, and 0.8 mg/L). Four shoots were cultured per Magenta box (Duchefa Biochemie, Haarlem, The Netherlands), with a total of 8 boxes per concentration/PGR combination.

In the RITA® TIS system, shoots were cultured on agar-free full-strength MS medium with 30 g/L sucrose, 0.1% PPM, and selected concentrations of PGRs, as described above. The explants were cultured following the manufacturer’s instructions (http://www.vitropic.fr (accessed on 19 April 2021)), with ten 2 cm long shoot explants per bioreactor. Shoots were temporarily immersed in 200 mL of liquid medium for 2 min every 4 h using an aerating pump.

The fresh weight of each shoot was approximately 18–20 mg, with a length of around 2 cm. In both systems, the pH was adjusted to 5.8, the temperature was maintained at 25 ± 2 °C, and a 16 h photoperiod was provided with a light intensity of 60–90 µmol photons m^−2^ s^−1^ (cool white fluorescent light). All media were autoclaved at 121 °C for 16 min.

At the end of each experiment, the number of shoots was recorded by counting the shoots and dividing that number by the number of initial explants in each Magenta box and bioreactor. Additionally, the shoot length and the relative growth rate (RGR) were calculated according Gatti et al. [51] as follows:

[Log_n_ fresh weight final − Log_n_ fresh weight initial] × 100/days of culture.

The cultivation of shoots with different PGR concentrations in both the SCM and RITA® TIS systems was repeated three times. The culture period was 4 weeks; every 4 weeks, shoot number per explant, shoot length, and RGR were recorded. The new shoots obtained after 4 weeks were used to prepare a new experimental replicate.

### 4.4. Rooting

For root induction, two different trials were conducted: in SCM and in rooting plugs (aseptic condition) with rockwool cubes, in which shoots were irrigated with Hoagland solution as a fertilizer. With the SCM, shoots 2 cm long with at least three properly formed leaves were cut and transferred to PGR-free ½ MS medium and ½ MS medium with one of three different concentrations (0.5, 1, or 2 mg/L) of indole-3-butyric acid (IBA). Shoots were cultured in Magenta™ GA-7 vessels with 60 mL of medium. With regard to rooting plugs, four rockwool cubes were placed in each Magenta vessel, saturated with Hoagland solution (approximately 20 mL), and then autoclaved. Finally, shoots were transferred to individual rockwool cubes. In both systems, shoots were incubated under a 16 h photoperiod (60–90 µmol photons m^−2^ s^−1^, cool white fluorescent light) at a constant 25 ± 2 °C for 5 weeks. For both experiments, 4 shoots per Magenta box were incubated, for a total of 8 Magenta boxes. After 5 weeks, the number and length of roots were determined.

### 4.5. Statistics and Data Analysis

Data were analyzed using R Studio (Version 2024.09.1+394) [52] and ImageJ [53]. The statistical significance of the effects of PGR concentration and culture system on shoot number, length, and RGR was assessed using a two-way analysis of variance (ANOVA). Differences between treatments were tested using Tukey’s post hoc test (*p* < 0.001).

Data analysis regarding the rooting experiment was performed as described above but using one-way ANOVA.

### 4.6. Plant Acclimatization

Rooted plants were transferred in a soil mix of peat and sand (3:1) in plastic pots (20 cm × 20 cm) and covered for the first 1 week with a plastic bag. Afterward, plantlets were grown uncovered in the greenhouse located in the Botanical Garden of Salento. After three months the number of surviving plants was recorded.

### 4.7. DNA Ploidy Level Determination After Protoplast Preparation and Nuclei Isolation

Protoplast preparation was carried out according to Di Sansebastiano and Barozzi [54] with some modifications. An amount of 450 mg of leaves from the mother plants and 150 mg from the regenerated-acclimatized plants of *V. ucriana* were cut and used for flow cytometry analysis.

After digestion, the leaves appeared to be digested, as they became morphologically translucent. However, no protoplasts were observed in the digestive solution, which was quite viscous due to the presence of mucilaginous compounds. Thus, under laminar flow, the digestive solution was removed from the Petri dish and replaced with W5 solution (9.0 g/L NaCl, 18.3 g/L CaCl_2_·2H_2_O, 0.37 g/L KCl, 1.0 g/L glucose) in order to allow the release of the protoplasts, which were trapped in the leaves. Leaves were gently stirred for a while with a plastic sterile pipette with a cut tip. The solution containing the protoplasts was then filtered with a 100–150 µm steel mesh (sterile) using a plastic sterile pipette with a cut tip. The previous steps were repeated twice, adding new aliquots of W5 to the leaves to increase protoplast recovery and dilute the mucilaginous compounds. At this point, filtered protoplasts were split into round-bottom tubes, with 5 mL of protoplasts in each tube. W5 solution was added on the protoplast solution up to 13 mL.

Before starting centrifugation, all tubes were slightly and carefully inverted to mix the solutions. Centrifugation was carried out at 65× *g* and 25 °C, without braking the swing-bucket rotor for 5 min.

Pelleted protoplasts from all tubes were pooled in two aliquots, and 13 mL of W5 solution was added. Before the second centrifugation, the solution was mixed gently by inversion. A second centrifugation was carried out under the same previous conditions (65× *g*, 5 min, no braking) in order to pellet the protoplasts again.

Centrifugations and washing steps with W5 were helpful in diluting/decreasing the amount of mucilaginous compounds, which negatively affected the next steps.

After centrifugation, supernatant was removed from pelleted protoplasts, and 1.5 mL of “nuclei isolation buffer” (200 mM Tris–HCl, pH 7.5, 4 mM MgCl_2_ × 6H_2_O, 0.5% *v*/*v* Triton X−100 supplemented with 1.5% (*w*/*v*) PVP − 10) was added. After 90 s, 200 µL of isolated nuclei was split into several Eppendorf tubes (1.5 mL) and stained with 10 µL of propidium iodide (initial concentration: 2 mg/mL).

*Arabidopsis thaliana* accession ‘Col-0’ (2C = 0.412 pg [55]) was used as the internal standard. Each sample was analyzed through a CytoFLEX S flow cytometer (Beckman Coulter, Indianapolis, IN, USA). Approximately 20,000 events were acquired for each sample and analyzed with CytExpert software (CytExpert 2.6.0.105). Debris and doublets were excluded based upon forward scatter and side scatter measurements. Three independent experiments were performed for the determination of the DNA ploidy level.

## Data Availability

Data are contained within the article.

## Supplementary Materials

The following supporting information can be downloaded at: https://www.mdpi.com/article/10.3390/plants13243530/s1, Figure S1: Germination percentage of *V. ucriana* seeds over time; Figure S2: Illustration of shoot development in different culture systems and with different PGR concentrations; Figure S3: Illustration of biomass grown in different culture systems and with different PGR concentrations; Figure S4: Number of roots developed in solid medium and rockwool; Figure S5: Root length in solid medium culture and rockwool; Figure S6: Locations of *V. ucriana* population where seeds were collected; Figure S7: Strategy applied for seed collection before sterilization and germination.
