# Supplementary material for: RITA® Temporary Immersion System (TIS) for Biomass Growth Improvement and Ex Situ Conservation of Viola ucriana Erben & Raimondo
Source: Plants (Basel). 2024 Dec 18;13(24):3530. doi: 10.3390/plants13243530 (PMC11676409; doi:10.3390/plants13243530)
Supplement: Supplementary file 1 [file plants-13-03530-s001.zip › plants-3363952-supplementary.pdf]

Article

RITA® Temporary Immersion System (TIS) for Biomass Growth Improvement and Ex Situ Conservation of *Viola ucriana* Erben & Raimondo

Piergiorgio Capaci [1], Fabrizio Barozzi [1], Stefania Forciniti [2], Chiara Anglana [1], Helena Iuele [2], Rita Annunziata Accogli [1], Angela Carra [3], Marcello Salvatore Lenucci [1], Loretta L. del Mercato [2] and Gian Pietro Di Sansebastiano [1,*]

Germination efficiency of *V. ucriana* germination *in vitro*

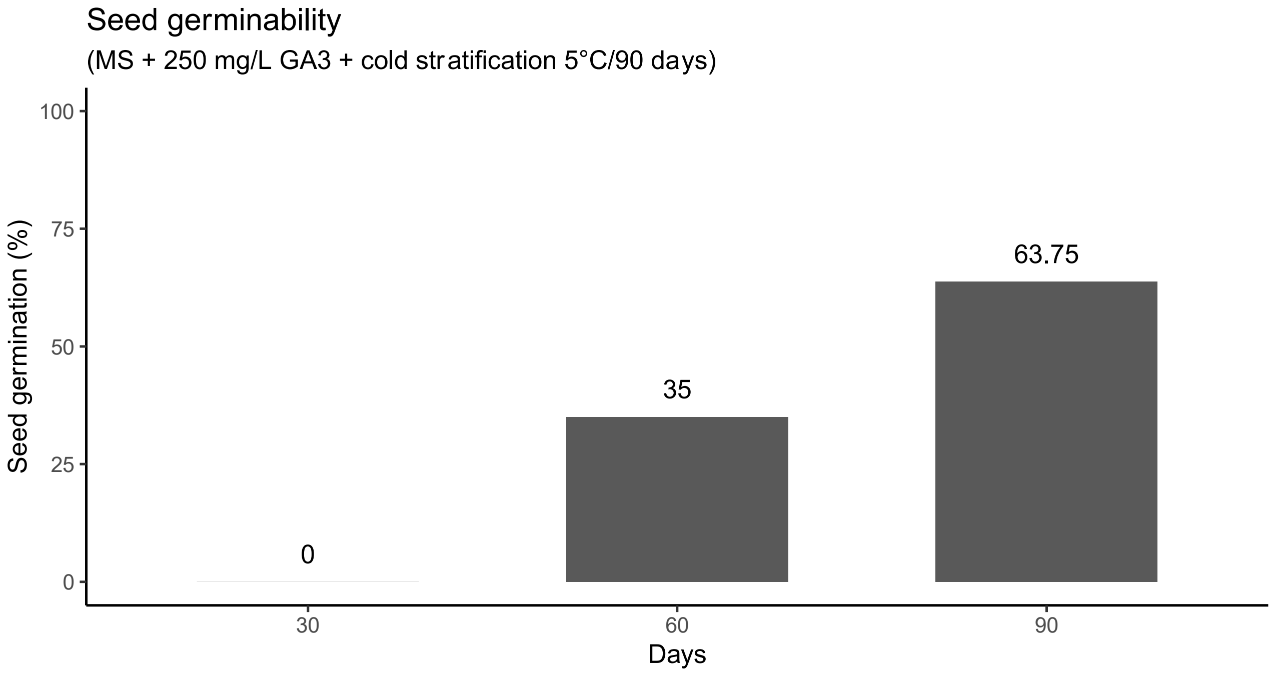

Figure S1. Percentage of *V. ucriana* seeds germination in time. Germination test was carried out applying GA3 on germination medium and transferring petri dishes in a cold room for cold stratification. Five petri dishes with 16 seeds for each were used for this purpose (total 80 seeds). Number of seeds germinated was recorder after 30, 60 and 90 days. Seed germination (%) was calculated as following: (number of seeds germinated after 30, 60 and 90 days / number of seeds collected) x 100

Shoots development diversified in different culture systems and with different PGRs concentration

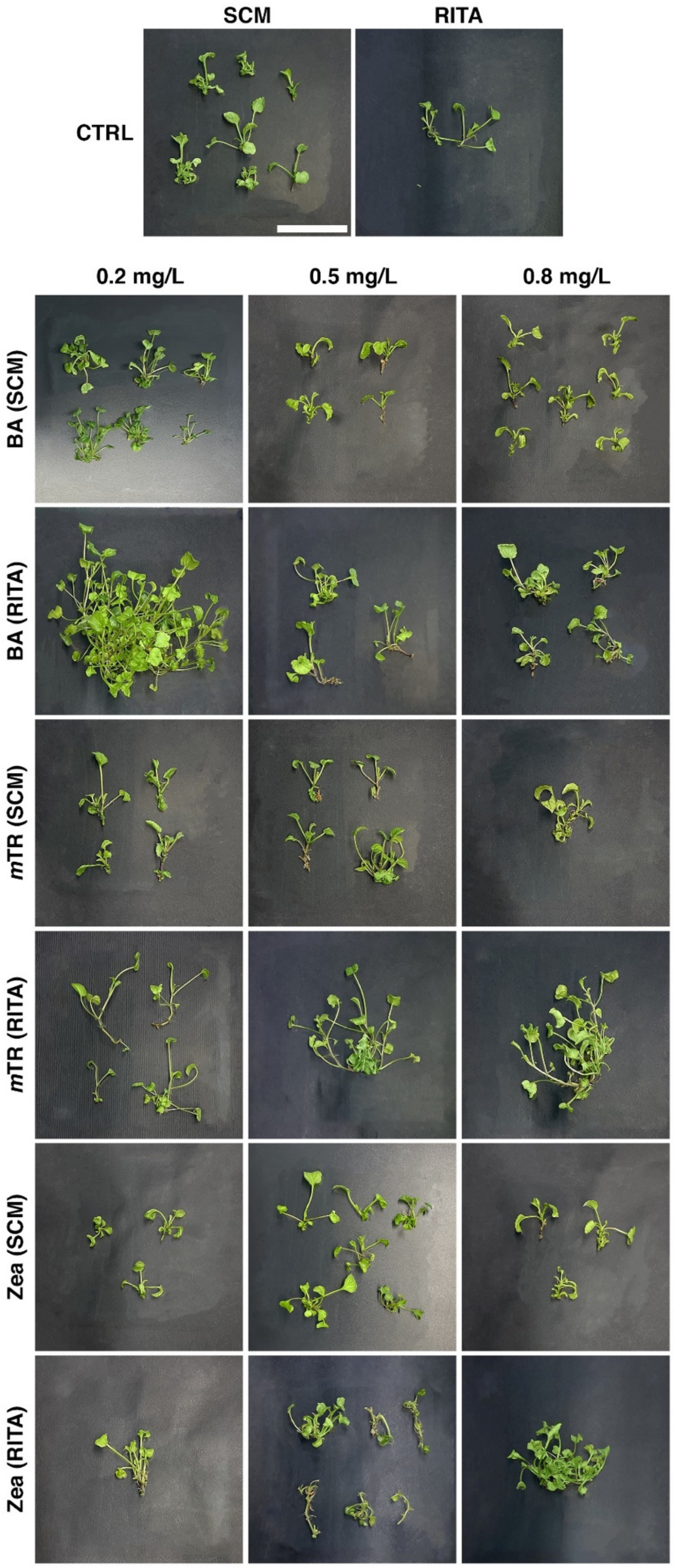

Figure S2. Illustration of shoots development in different culture systems and different PGRs concentration. Shoots were grown in different culture systems (solid medium culture: Magenta box and temporary immersion system: RITA bioreactor) and PGRs concentration (BAP: 0.2 – 0.5 – 0.8 mg/L; *m*TR: 0.2 – 0.5 – 0.8 mg/L; Zeatin: 0.2 – 0.5 – 0.8 mg/L). Scale bar = 25 mm.

General appearance of biomass in different culture systems and with different PGRs concentration

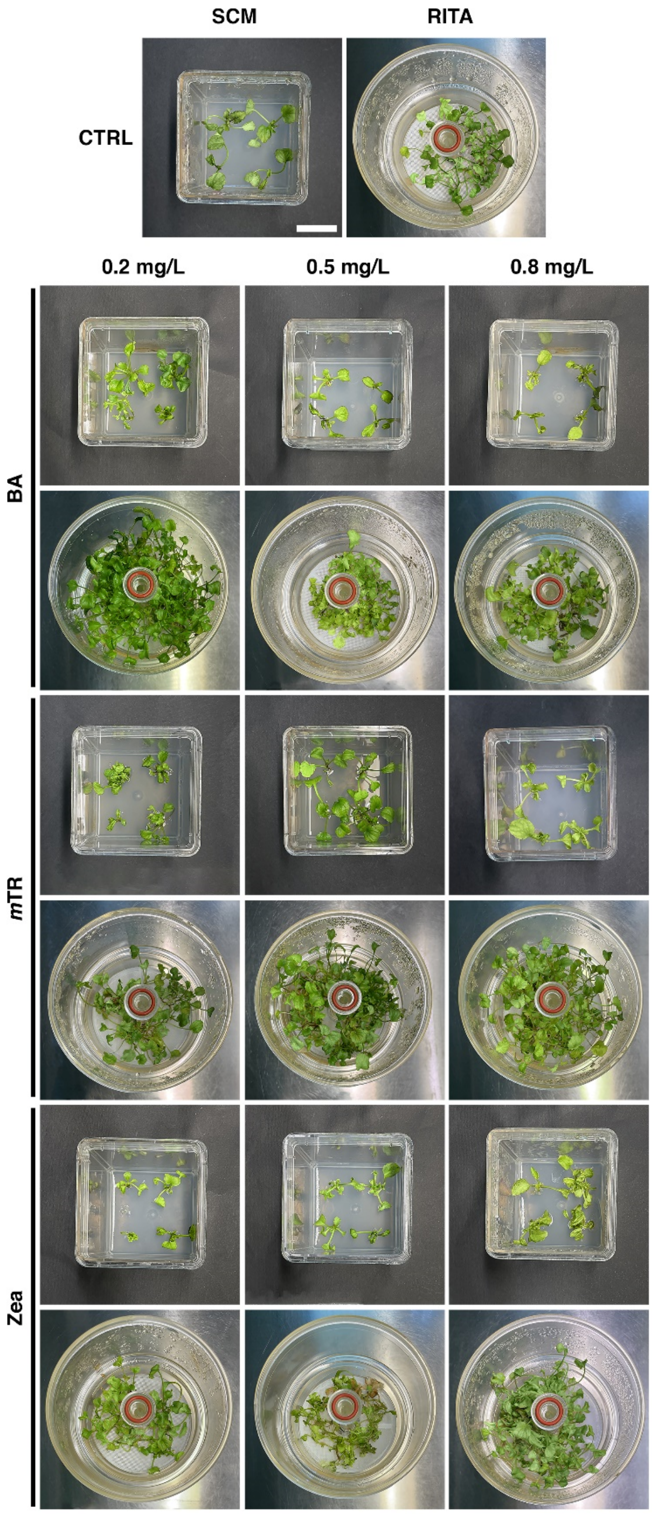

Figure S3. Illustration of biomass grown in different culture systems and with different PGRs concentration. Biomass were grown in different culture systems (solid medium culture: Magenta box and temporary immersion system: RITA bioreactor) and PGRs concentration (BAP: 0.2 – 0.5 – 0.8 mg/L; *m*TR: 0.2 – 0.5 – 0.8 mg/L; Zeatin: 0.2 – 0.5 – 0.8 mg/L). Scale bar = 25 mm.

Number of roots developed in solid medium culture and rock wool

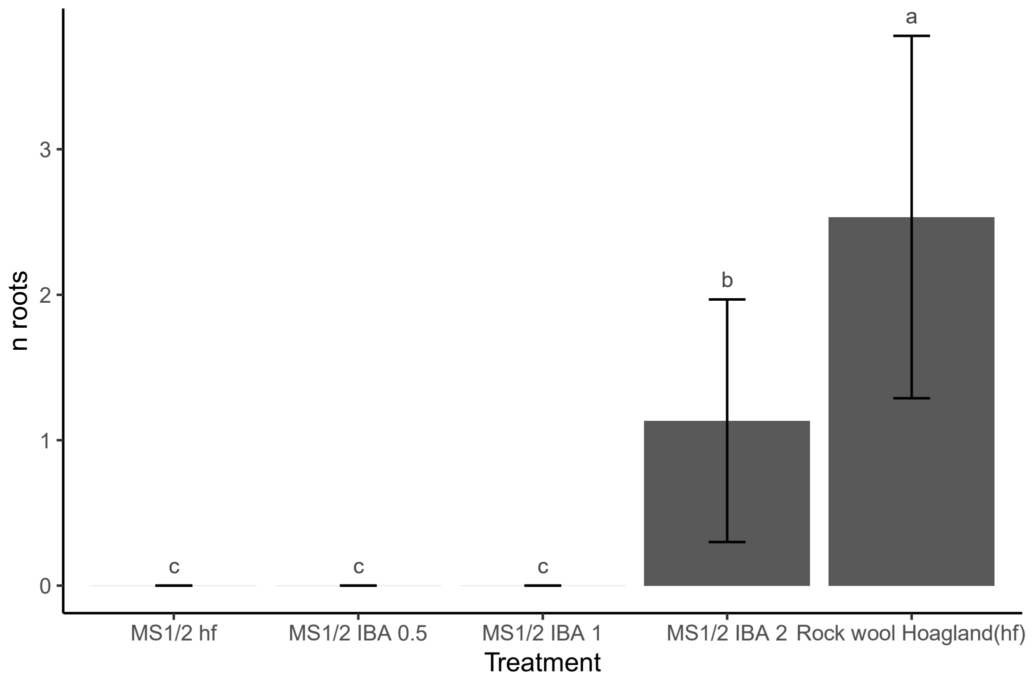

Figure S4. Number of roots developed in solid medium culture and rock wool. Differences on number of roots emerged in solid medium culture (MS ½ hormone free and MS ½ with 0.5, 1, 2 mg/L of IBA) and in rock wool.

Roots length developed in solid medium culture and rock wool

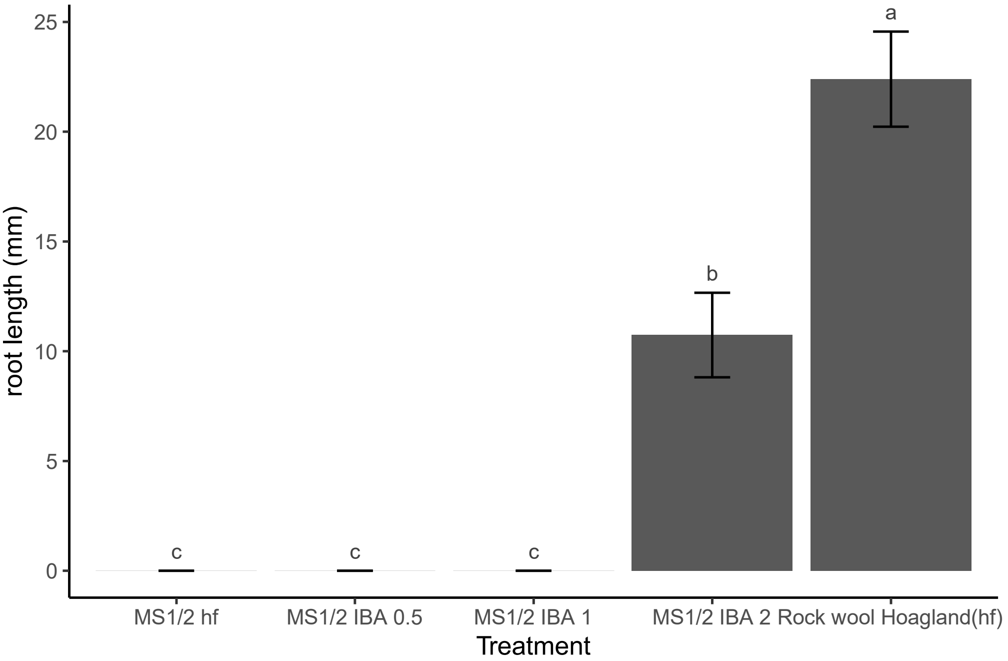

Figure S5. Roots length developed in solid medium culture and rock wool. Differences on roots length emerged in solid medium culture (MS ½ hormone free and MS ½ with 0.5, 1, 2 mg/L of IBA) and in rock wool.

Location of *V. ucriana* plants population.

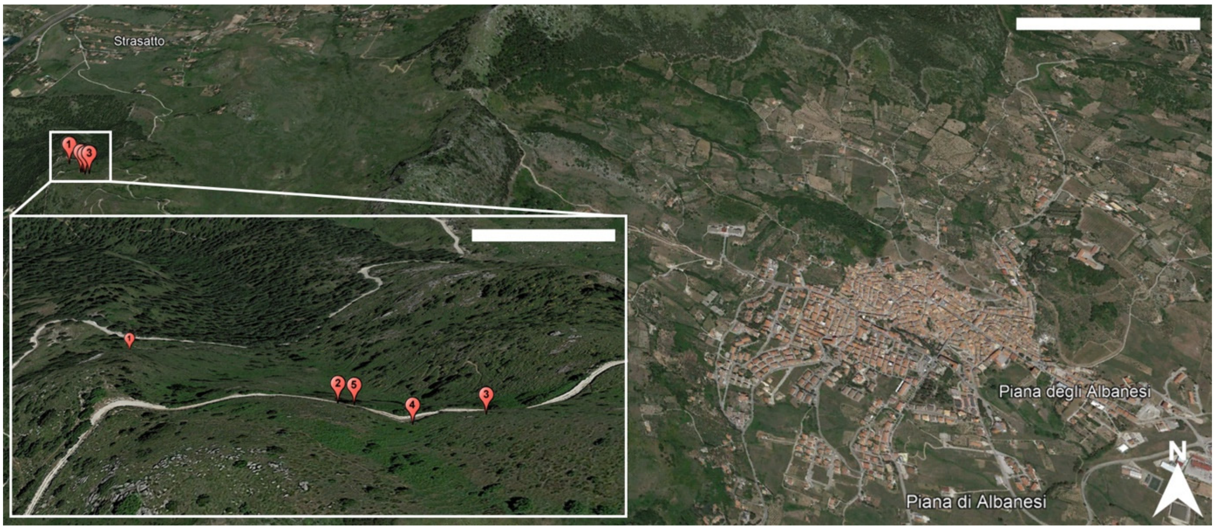

Figure S6. Location of V. ucriana population where seeds were collected. Seeds were collected in Fratantoni slopes, near the Pizzuta top in Piana degli Albanesi (NW Sicily, Italy). Seed collection involved five different sites: 1) N38°00′00.15″ E 13°15′00.68″, alt 1002 m; 2) N37°59′56.10′ E13°15′04.87″, alt 1077 m; 3) N37°59′55.25″ E13°15′06.56″, alt 1094 m; 4) N37°59′55.51″ E13°15′05.77″, alt 1083 m; 5) N37°59′56.20″ E 13°15′05.08″, alt 1074 m. Different places were chosen in order to generate from seeds different clonal lines with genetic variability, somehow representative of the genic pool of the whole population.

Fruit and seeds of *V. ucriana*.

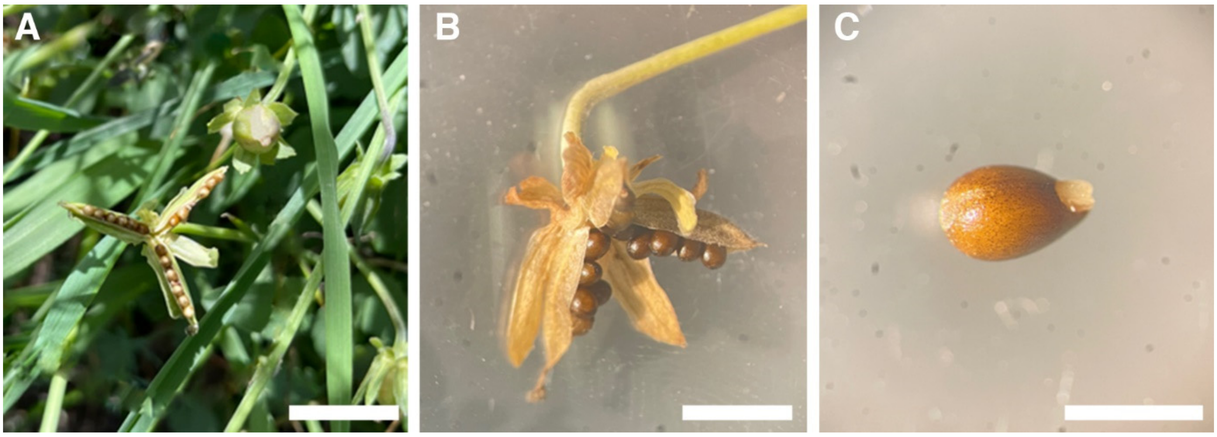

Figure S7. Strategy applied for seed collection before sterilization and germination. (**A**) fruit split open ready to be disperse ballistically (to the left) and fruit pointed upward ready to be collected (to the right). (**B**) opened capsule in a controlled space with mature seeds. (**C**) *V. ucriana* tipically brown seed with its drop shape. Scale bars **A** = 10 mm, **B** = 5 mm, **C** = 2 mm
